# Research Progress on the Enhancement of Immobilized Enzyme Catalytic Performance and Its Application in the Synthesis of Vitamin E Succinate

**DOI:** 10.3390/molecules30061241

**Published:** 2025-03-10

**Authors:** Liang Qu, Qiongya Lu, Liming Zhang, Fanzhuo Kong, Yuyang Zhang, Zhiyuan Lin, Xing Ni, Xue Zhang, Yani Zhao, Bin Zou

**Affiliations:** 1 School of Food and Bioengineering, Wuhu Institute of Technology, Wuhu 241003, China; 101385@whit.edu.cn; 2 School of Food and Biological Engineering, Jiangsu University, Zhenjiang 212013, China; 2222418088@stmail.ujs.edu.cn (Q.L.); 3046077379@stmail.ujs.edu.cn (L.Z.); 2222218033@stmail.ujs.edu.cn (F.K.); 2212218043@stmail.ujs.edu.cn (Y.Z.); 2222218077@stmail.ujs.edu.cn (Z.L.); 2212318069@stmail.ujs.edu.cn (X.N.); 2212318042@stmail.ujs.edu.cn (X.Z.); 2212418062@stmail.ujs.edu.cn (Y.Z.)

**Keywords:** immobilized lipase, nanomaterials, vitamin E succinate, enzymatic catalysis

## Abstract

Vitamin E succinate is a more mature vitamin E derivative, and its chemical stability and many effects have been improved compared with vitamin E, which can not only make up for the shortcomings of vitamin E application but also broaden the application field of vitamin E. At present, in developed countries such as Europe, America, and Japan, vitamin E succinate is widely used in health foods, and due to its good water solubility and stability, the vitamin E added to most nutritional supplements (tablets and hard capsules) is vitamin E succinate. At the same time, vitamin E succinate used in the food and pharmaceutical industries is mainly catalyzed by enzymatic catalysis. In this paper, *Candida rugosa* lipase (CRL) was studied. Chemical modification and immobilization were used to improve the enzymatic properties of CRL, and immobilized lipase with high stability and high activity was obtained. It was applied to the enzymatic synthesis of vitamin E succinate, and the reaction conditions were optimized to improve the yield and reduce the production cost. The review covered the research progress of the methods for enhancing the catalytic performance of immobilized enzymes and discussed its application in the synthesis of vitamin E succinate, providing new ideas and technical support for the catalytic performance enhancement of immobilized enzymes and its application in the synthesis of vitamin E succinate and promoting the production and application of vitamin E succinate.

## 1. Introduction

There are many types of enzymes, and depending on the type of catalyzed reaction, enzymes can be divided into the following six categories: oxidoreductases, hydrolases, transferases, lyases, isomerases, and ligases [1,2,3,4,5]. Among them, lipase is an important hydrolase enzyme, which is widely used in the study of biocatalysis because of its ability to catalyze reactions with less by-product production, low waste disposal cost, and mild reaction conditions [6,7,8,9].

### 1.1. Overview of Lipases

Lipase (EC 3.1.1.3) is widely found in plants, animals, and microorganisms; it is a special class of acylhydrolases [10]. In the aqueous phase, it catalyzes the hydrolysis of its native substrate, triglycerides, to produce mono- or diacylglycerides, glycerol, and free fatty acids [11]. In the non-aqueous phase (organic solvent or supercritical fluid), organic substances such as esters, acids, alcohols, anhydrides, and amides can be used as substrates to catalyze the reactions of esterification, transesterification, alcohololysis, acidolysis, and aminolysis [12,13,14,15,16].

### 1.2. Lipase Catalytic Mechanism

**Figure 1:**
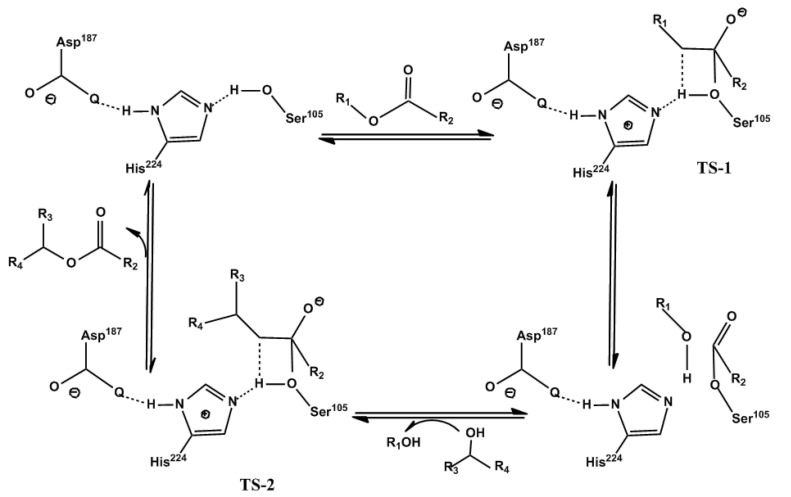
Mechanism of lipase-catalyzed esterification or hydrolysis.

The lipase molecule is composed of the following two parts: the hydrophilic end and the hydrophobic end. And the active center is located at the hydrophobic end. Sources of lipase are different; the amino acid composition is also different, but the structure of the active center is the same or similar, that is, the catalytic triad composed of histidine, serine, and aspartic acid or glutamic acid. In general, the active center of lipase is replaced by a helical polypeptide chain (“lid”) overlay. “Lid” will protect the active center of lipase, which can be found in “closed” and “opening” toggles between conformations [14]. When the substrate is not present, the “lid” is in a closed state, and after the substrate is added, the conformational cause of the lipase electrophilic domain role. When the “lid” is opened, revealing its active center, the substrate enters and binds to it to allow the enzyme to act catalytically. This process is related to the conformational change of the enzyme molecule, which leads to the reorientation of the oxygen anion hole, which has been confirmed by X-ray crystallo graphy of *Rhizomucor miehei* lipase [14]. Lipase-catalyzed hydrolysis reactions in the opposite direction follow a ping-pong mechanism (Figure 1). The recombination of the three amino acids in the active center of the enzyme increases the nucleophilic ability of the tryptophan residue, which is conducive to further attacking the carbonyl group of the acyl group donor and forming an “acyl–enzyme complex”. Then, the substrate will further attack the acyl–enzyme complex to produce the product [17,18].

### 1.3. Application of Lipase and Its Existing Problems

(1) Applications in the food industry

In the food industry, lipase is widely used in oil processing, dairy production, pasta processing, and meat processing [19,20,21,22,23]. Oils and fats are important components of food, and changing the molecular structure of fatty acids can improve the nutritional value of oils. Using the specificity of lipase, it can selectively hydrolyze specific ester bonds in oils and fats to produce high value-added oils or for the synthesis of polyunsaturated fatty acids and the modification of special oils [24,25]. At present, lipase has been used to replace the traditional catalyst sodium methoxide to catalyze the esterification reaction to synthesize glycerides, which can be used in the production of a variety of dairy products such as cheese, milkshakes, and cream [26]. Lipase can also be used to mature cheese and promote the development of special flavors in foods, while lipase-treated dairy products have better flavor and aroma. In the process of producing pasta food, adding an appropriate amount of lipase can also improve the elasticity of the dough, making the dough transparent and textured and not easy to break and improving the taste of the dough.

(2) Application in the chemical industry

In the production process of chemical products, the use of lipase as a catalyst can reduce the production of by-products in the reaction, thereby reducing the production cost; the polymer materials synthesized with enzymes as catalysts are also easy to degrade by microorganisms, and the intermediate products and wastes produced in the chemical synthesis process are easy to cause environmental pollution and other problems [27,28]. Enantiomeric resolution of chiral intermediates is required in the synthesis of commonly used pesticides such as herbicides, insecticides, and fungicides. The chemical resolution method has a high cost, high price, high catalyst toxicity, unfavorable operation, difficult product separation, low yield, and easy pollution of the environment. In contrast, the enzyme-catalyzed reaction is easy to operate, has a high yield, fewer by-products, and is environmentally friendly [29,30]. Therefore, the application of lipase as a catalyst in the production of chemical products is an important research direction for the development of “green” chemical industry at present.

(3) Application in textiles

The application of lipase in the weaving and dyeing process can enhance the binding force between the fabric and the dye so that the color of the fabric and dyeing is more uniform and the color is maintained more durably [7]. Lipase can also reduce the grease in the fabric raw material and make it meet textile requirements without compromising the strength of the fiber. Lipase can also be used to remove some of the fat attached to the fur, making the fur product more elastic and soft, without affecting the thickness and fastness of the leather [31].

(4) Application in medicine and health

Lipase catalysis has the characteristics of stereoselectivity and high regioselectivity, which makes it an important biocatalyst for the preparation of chiral drugs. Intermediates of drugs such as paroxetine, ibuprofen, and metoprolol can be synthesized by lipase [32,33]. Penicillium TS414 lipase has good enantiomeric selectivity and catalytic activity against naproxen and is often used as a tool enzyme for the resolution of racemic naproxen. *Aspergillus niger* lipase hydrolysis of racemic ethyl ester prepares R-isomer, which is the main component of serotonin antagonists, an anti-arrhythmia drug. Paroxetine [32] and other statin side-chain hypolipidemic drugs [34]. It can also be synthesized by microbial lipase. In addition, lipase can also be used to treat diseases such as pancreatitis, hyperlipidemia, dyspepsia, and malignant tumors.

(5) Application in environmental protection

The application of lipase in environmental protection is a research direction that has gradually emerged in recent years [35]. It is mainly reflected in the enzymatic treatment of waste and the enzymatic synthesis of new energy. Lipase can be used to treat fatty waste from oil processing and edible waste oil from catering [36]. Replacing chemicals added to detergents with lipase avoids secondary pollution to the environment and reduces disposal costs [37], and the detergent with enzyme preparations can still maintain a good decontamination effect under low-temperature conditions [38]. Lipase can also be used as a biocatalyst to catalyze the transesterification of oils and fats with short-chain alcohols to synthesize biodiesel [39]. Enzymatic synthesis of biodiesel requires low raw materials and mild reaction conditions and is environmentally friendly, which is a new trend in the development of synthetic biodiesel.

With the improvement in people’s living standards, consumers pay more and more attention to whether the ingredients added in food, cosmetics, and pharmaceutical products are natural ingredients, and the market demand for natural ingredients will be greatly improved in the future.

Among them, CRL is a Sn-1/3 specific lipase, which has excellent enzymatic properties and can catalyze a variety of reactions, such as esterification, transesterification, hydrolysis, and chiral resolution, and be used in different industrial fields according to the catalytic characteristics of different isozymes. Srivastava et al. used CRL1 to catalyze the transesterification of tripalmitic acid with oleic acid (or methyl oleate) to generate a human milk fat substitute [40]. Su et al. used jatropha seed oil as a raw material and CRL2 as a catalyst to catalyze the synthesis of biodiesel [41]. Ivić et al. used commercial CRL as a catalyst to catalyze the esterification of coconut oil and vanillyl alcohol to produce capsaicin esters [42]. Hu et al. catalyzed the synthesis of vitamin E succinate by interfacial activation of CRL embedded in sol–gel [43]. Vorlová et al. used the specific properties of CRL1 to selectively hydrolyze D, L-benzoate to produce L-menthol [44]. These methods use CRL as a catalyst to achieve remarkable results in different catalytic reactions, demonstrating the wide application and importance of CRL in catalytic reactions. The results showed that CRL had good catalytic performance [45].

However, the chemical nature of lipase is protein, and the direct application of free lipase as a catalyst in industry will face a series of problems. Free lipase is not stable enough in extreme environments such as organic solvents, high temperature, high pressure, strong acid, and strong alkali, and its activity is easy to lose, and its main disadvantages can be summarized as follows [46,47,48,49]:

Free lipase itself is prone to agglomeration, resulting in inactivation or degradation, and the utilization rate of the enzyme is low.

Free lipase has poor stability and is inactivated at high temperatures and strong acid–base conditions.

Free lipases are difficult to separate from substrates and products, affecting the purity and quality of products, and it is difficult to achieve continuous and automated production.

The catalytic performance of free lipase is easily affected by other factors such as substrates and products in the process of participating in the reaction.

Free lipase is difficult to recover and difficult to reuse, resulting in increased production costs.

Due to the problems of free lipase in catalytic reactions, it is difficult to realize the industrial application of free lipase. In order to solve these problems, enzyme immobilization technology came into being. Immobilized enzymes not only have the catalytic characteristics of enzymes but also can be recycled and reused like general chemical catalysts and realize the continuity and automation of the production process.

## 2. Immobilized Lipase

### 2.1. Application of Immobilized Lipase in the Synthesis of Vitamin E Succinate

Vitamin E succinate is an important vitamin E derivative that has better chemical stability and multiple physiological effects than vitamin E. At present, the synthesis of vitamin E succinate mainly adopts the following two methods: chemical catalysis and enzyme catalysis. Compared with chemical catalysis, enzyme catalysis has the advantages of mild reaction conditions, high catalytic efficiency, and strong catalytic specificity, but the catalytic efficiency still needs to be improved.

Immobilized lipase has a broad application prospect in the synthesis of vitamin E succinate. Through chemical modification and immobilization technology, the catalytic activity and stability of lipase can be improved so as to increase the yield of vitamin E succinate and reduce the production cost.

### 2.2. Preparation Method of Immobilized Lipase

At present, the commonly used methods of lipase immobilization include physical fixation (adsorption and embedding) and chemical fixation (covalent and cross-linking method) (Table 1) [50,51,52].

The adsorption method is through non-specific adsorption with the carrier’s direct adsorption and fixation of enzyme molecules on the carrier. According to the different forces, it can be divided into the physical adsorption method and the ion exchange adsorption method [53].

The physical adsorption method is the van der Waals force between the enzyme molecule and the carrier, electrostatic action, and physics such as hydrophobic bonds and hydrogen bonds. The force will be the enzyme molecule’s direct adsorption and fixation on the carrier [54]. This method is easy to operate, the reaction conditions are mild, and the active center and tertiary structure of the enzyme molecule can be well protected, and the recovery rate of enzyme activity is high. However, the interaction between the enzyme and the carrier is weak, resulting in easy detachment of enzyme molecules from the carrier.

Shinji et al. combined the effects of *Pseudomonas cepacia* lipase (PCL) fixed on electrospun polyacrylonitrile (PAN) fibers by physical adsorption and used it to catalyze the production of glycidyl n-butyrate [55].The results showed that the reaction rate of immobilized enzyme was 23 times higher than that of the initial material, and the reaction rate was still 80% of the initial reaction rate after 10 cycles. Li et al. used *porcine pancreas* lipase (PPL) that was immobilized on rod-shaped SBA-15, and the effects of different pH and different reaction times on enzyme immobilization were studied. The results showed that pH 6.0 and 3 h reaction were the best immobilization conditions; the enzyme activity of the immobilized enzyme obtained was the highest, and the thermal stability of immobilized PPL was significantly improved compared with that of free PPL [56].

The ion exchange adsorption method, also known as the ion binding method, refers to the immobilization of enzyme molecules on the support through the electrostatic force between the side chain dissociation group on its surface and the ion exchange group of the carrier under suitable ionic strength and pH conditions. The ion exchange adsorption method is simple to operate, mild to the conditions, and not easy to damage the structure of the enzyme while retaining the high activity of the enzyme molecule.

Cai et al. used macroporous adsorption resin AB-8 as the carrier to immobilize and expand Penicillium TS414 lipase by the ion exchange adsorption method, which significantly improved the dispersion of lipase in the organic phase reaction medium and improved the efficiency of the enzymatic resolution reaction. Adi et al. immobilized *Candida antarctica* lipase on acrylic resin by the ion exchange adsorption method, and the immobilized enzyme obtained was used to catalyze the reaction of triacetate with isoamyl alcohol to synthesize isoamyl acetate. The yield can reach more than 80%, and the immobilized enzyme retains high yields after 4 reuses [57].

The embedding method is to mix the enzyme solution with the monomer of the polymer, and the monomer is polymerized under the action of the polymerization initiator, and the enzyme molecule is embedded in the microcapsule structure or lattice structure of the carrier. This allows the substrate to enter the lattice and come into contact with the enzyme molecules without the enzyme protein shedding. The method is relatively simple to operate, and at the same time, the enzyme molecule is only embedded, and the amino residues on its surface are not bound to react, and the spatial conformation is changed to a lesser extent, so its biological activity is reduced. It can be retained to a large extent [58,59].

Monier et al. fixed CRL, which showed that the immobilized enzyme had improved thermal stability and affinity for substrates and maintained 75% biological activity after 6 repeated uses [60]. Raman et al. combined *Burkholderia cepacia* lipase (BCL) that was embedded in carrageenan, and its enzymatic properties were studied. The results showed that the stability of the immobilized enzyme (pH, heat, and storage), reusability, and resistance to organic solvents were improved, and the activity of the immobilized enzyme was still 72% after 6 repeated uses [61].

The covalent binding method is a method in which the functional groups on the surface of the enzyme are covalently coupled with the carrier groups to form an immobilized enzyme. In this method, the binding force between the enzyme molecule and the carrier is relatively firm, and the enzyme molecule is not easy to fall off when used, so as to improve the stability of the enzyme. However, the disadvantage of this method is that the activation or immobilization of the carrier is complicated, and the covalent binding may affect the active site of the enzyme, resulting in the denaturation of the enzyme molecule, resulting in the decline of enzyme activity.

Chiou et al. used chitosan-activated carbodiimide to fix CRL and studied the enzymatic properties of the immobilized enzyme, and the results showed that the acid–base stability and thermal stability of the immobilized enzyme were improved compared with the free enzyme, and 85% of the residual enzyme activity was retained after 10 repeated uses [62]. Liu et al. fixed BCL on diatomite modified with 3-aminopropyl triethoxysilane using glutaraldehyde as a cross-linking agent, and the thermal stability of the immobilized enzyme was significantly improved, and the residual activity of the immobilized enzyme was 83% after holding at 55 °C for 2 h, which was twice that of the free enzyme [63]. Liu et al.’s method is more prominent in terms of thermal stability and may be more suitable for reactions that need to be carried out at higher temperatures. However, Chiou et al.’s method performs well in acid–base stability and reusability and may be better suited for applications that require a wider pH range or multiple reuses.

The cross-linking method uses multifunctional reagents (glutaraldehyde, k-carrageenan, etc.) as cross-linking agents to make enzyme molecules bound together through covalent bonds; thus, a three-way cross-network frame structure is formed, and this structure is relatively stable. Meantime, according to the different materials and use conditions, immobilized enzymes with different properties can be obtained [64]. There are about three ways to prepare immobilized enzymes by multifunctional reagents as follows: (1) interaction with enzymes alone; (2) the enzyme is first adsorbed on the surface of the carrier and then cross-linked; (3) the functional group is introduced on the surface of the carrier through a multifunctional reagent and then linked to the enzyme. The immobilized enzyme prepared by the cross-linking method has a firm binding of enzyme molecules to the carrier, is not easy to fall off, and has high stability. However, in this method, the functional groups of the enzyme protein molecule are involved in the reaction, and the loss of enzyme activity is large. Therefore, in practical applications, the cross-linking method is rarely used alone and is often combined with the adsorption method, the embedding method, and other methods to achieve better results.

Gao et al. used glutaraldehyde as a cross-linking agent and made the enzyme and chitosan form a stable network structure covering the surface of the carrier through the “surface cross-linking” method. The results showed that the stability of the immobilized enzyme was significantly improved, and 85% of the enzyme activity was maintained after 6 repeated uses [65]. Raman et al. used k-carrageenan as a cross-linker to immobilize BCL, and the embedding rate reached 42.6%, and the immobilized enzyme retained 72.3% of the initial enzyme activity after 6 repeated uses [61]. In summary, if a highly stable immobilized enzyme is required, the method of Gao et al. can be selected. If immobilized enzymes with high enzyme activity are required, Jegannathan et al.’s method can be chosen.

### 2.3. Carriers for Immobilized Lipases

With the rapid development of enzyme immobilization technology, many practical immobilization materials have been discovered, and at present, the commonly used carrier materials can be divided into the following two categories: traditional carrier materials and new carrier materials [66,67].

Traditional carrier materials are mainly inorganic carrier materials and natural polymers. Commonly used inorganic carrier materials are activated carbon and porous glass, silicon oxide, molecular sieves, etc. They all have the advantage of being low cost. It is characterized by high mechanical strength and good stability. However, their small specific surface area and pore size and large particle size limit their application [68]. Natural polymer materials refer to natural polymer compounds, such as polysaccharides, proteins, and other substances. The new carrier materials mainly include organic–inorganic composite materials, synthetic polymer materials, and nanomaterials [69,70,71,72]. Organic–inorganic composite materials refer to the combination of polymer materials and inorganic materials by a certain method to form a new carrier material [73,74,75]. Mahmoud et al. combined functionalized cellulose nanocrystals (CNCs) and gold nanoparticles (AuNPs) to form novel CNC/AuNPs materials for enzyme immobilization [76]. Synthetic polymer materials have high mechanical strength, anti-microbial degradation properties, and low cost. At present, the use of nanostructured materials as carriers has become a turning point in the field of immobilized enzymes. Nanostructured materials have the advantages of large specific surface area, low mass transfer resistance, high enzyme loading, and good enzyme-catalyzed microenvironment [77,78,79,80]. As an immobilized carrier material, it has a good application prospect.

## 3. Nanomaterial Immobilized Lipases

In general, nanomaterials commonly used as immobilized lipase carriers include nanoporous materials and organic polymer nanomaterials.

### 3.1. Nanoporous Materials-Metal-Organic Frameworks (MOFs)

Metal-organic frameworks (MOFs) are crystalline nanomaterials. It has a variety of structures and functions; the nanopores are ordered, and the hydrophilicity and hydrophobicity are adjustable [81,82,83,84,85,86,87]. The use of MOFs as immobilized enzyme carriers can better maintain the catalytic activity of enzymes, reducing the mass transfer resistance of immobilized enzymes in catalytic reactions, and improve the stability of enzyme molecules [88,89]. However, not all MOFs are suitable for immobilized lipase, and the selection of suitable MOF carriers should consider the pore size, shape, hydrophilicity, chemical stability, and other factors. The commonly used MOF carriers, such as ZIF-8 and Fe_3_O_4_@MOF, have good application prospects in immobilizing lipase. Nadar et al. immobilized Aspergillus niger lipase on ZIF-8 by the biomineral method, and the thermal stability and reuse stability of the immobilized enzyme were improved, nd 54% residual activity was retained after 7 consecutive reuses [90]. Wang et al. fixed the CRL on the Fe_3_O_4_@MOF. The results showed that CRL-Fe_3_O_4_@MOF 65% of the residual activity was maintained after incubation at 65 °C for 6 h, and after 10 repeated uses, the residual activity was still about 60% [91]. Li et al. used zinc acetate and adenine as metal ions and organic ligands and successfully immobilized thermophilic lipase in MOFs by the biomimetic mineralization method, and the results showed that the enzyme loading rate of the immobilized enzyme was 15.9%, and the hydrolysis of p-nitrophenyl octanoate was used as a model, which proved that the immobilized enzyme could still maintain good catalytic activity under high temperature and alkaline conditions and in the presence of metal ions [92].

### 3.2. Organic Polymer Nanomaterials

Organic polymer nanomaterials can be applied to a variety of enzyme immobilization and enzyme catalytic systems due to their diverse structures, ordered pores, and adjustable hydrophobicity [93,94]. The nanostructured enzyme catalysts prepared with organic polymer nanomaterials as carriers not only maintain the catalytic activity of the enzyme, improve the high-temperature resistance and corrosion resistance of the enzyme, and reduce the mass transfer resistance of the enzyme-catalyzed reaction but also facilitate the recovery of enzymes [95]. Danial et al. exploit the electrostatic interaction between anionic polymers and block cationic polymers; the micelle carrier was synthesized, and then the enzyme composite micelles were prepared by the combination between the aldehyde functional group on the surface of the micelle carrier and the amino group on the surface of the enzyme molecule [96]. Jia et al. immobilized the enzyme on the surface of polystyrene nanofibers. In total, 65% of the native enzyme activity is retained; compared to natural enzymes, its half-life in methanol is increased by a factor of 18 [97].

### 3.3. Preparation Method of Nanostructured Enzyme Catalyst

Enzyme catalysts with nanostructures can be formed by immobilizing enzyme molecules on the surface of nanomaterials or embedding them within the nanomaterial structure. Based on the synthesis strategy, the preparation methods of nanostructured enzyme catalysts can be divided into the following three categories.

(1) Methods based on surface fixation of nanomaterials

The nanomaterials are synthesized and prepared, and then, the enzymes are immobilized on the surface of the nanomaterials by traditional immobilization methods. In this method, the specific surface area of the nanostructure is larger, which can increase the amount of enzyme loading. At the same time, different methods can be selected to immobilize the enzyme on the surface of the carrier according to the different catalytic reaction systems. In addition, the surface properties of nanomaterials can also be designed according to different reaction systems so as to improve the preparation efficiency of nanostructured enzyme catalysts and the performance of immobilized enzymes. Huang et al. modified the surface of ceramic foam with silane coupling agent and fixed penicillium alkaline lipase on the surface. The results showed that the immobilization rate of lipase was increased, and the thermal stability of the immobilized enzyme was also significantly improved [98]. Shinji et al. immobilized Pseudomonas cephalomonas lipase on polyacrylonitrile (PAN) electrospun fibers and converted (S)-glycidyl with vinyl butyrate to glycidyl butyrate in isooctane. The reaction rate of immobilized lipase was increased by a factor of 23, and 80% of the initial reaction rate was maintained after 10 reuses [55].

(2) Methods based on the assembly of nanomaterials

During the nanomaterial assembly process, enzyme molecules are embedded in a carrier. In this method, the structures of nanomaterial carriers are diverse, and their structures are designable so that nanostructured enzyme catalysts can be adapted to a variety of enzyme molecules and catalytic systems. Say et al. prepared lipase nanoparticles by embedding lipase in nanomaterials by microemulsion polymerization technology. The results showed that the lipase nanoparticles exhibited high catalytic activity in the range of pH 6.0 to 10.0 and were easily separated from the reaction for reuse [99]. Velonia et al. embedded CALB in polystyrene by assembling enzymes and polymers into nanowires, and the nanostructured enzyme catalyst still retained about 70% of enzyme activity [100].

(3) Methods based on in situ synthesis of block copolymers

The covalent binding method is combined with the embedding method, and the enzyme molecule is chemically modified first; it is then embedded in nanomaterials through covalent bonds in the organic or aqueous phases, that is, the method of in-situ synthesis. The in-situ synthesis method has a high yield of more than 80%, which is mainly because the in-situ binding reaction occurs between the enzyme molecule and the small monomer molecule. At the same time, the connection between nanomaterials and enzyme molecules and the suitable microenvironment in nanomaterials help to improve the stability of enzyme molecules and the resistance to organic solvents [101,102]. Maynard et al. chemically modified the enzyme molecule, introduced the polymerization initiator molecule, and prepared the polymer-modified enzyme in the aqueous phase, which maintained the activity of the enzyme well [103].

At present, there are many studies on the first and second methods, and the research on the third method has gradually increased in recent years.

## 4. Research Progress on the Directional Immobilization of Biological Enzymes in Nanomaterials

The fixation of various enzymes by different carbon nanomaterials and different magnetic nanomaterials at home and abroad is summarized in Table 2. With the advancement of nanotechnology, nanomaterials have been widely used in many fields such as medicine, pharmacy, chemistry, environmental monitoring, and microbial fuel cells. Carbon nanomaterials, including carbon nanotubes (CNT), graphene G, and its derivatives (GO and rGO), are commonly used immobilized enzyme materials with excellent physical and chemical properties. Among magnetic nanomaterials, Fe_3_O_4_ is a commonly used magnetic nanomaterial, which can be used to immobilize enzymes and recover them through an applied magnetic field. The results showed that both carbon nanomaterials and magnetic nanomaterials could significantly improve the activity and stability of enzymes. Different materials and immobilization methods have different immobilization effects on enzymes, and appropriate materials and methods should be selected according to specific applications. Lipase, as the most commonly studied enzyme, is widely used in biodiesel production, food processing, and other fields. Both carbon nanomaterials and magnetic nanomaterials can significantly improve the activity and stability of lipase.

**Table 2:** Summary of research progress of different nanomaterials for enzyme fixation.

Immobilized Vectors	Enzyme Species	Fields of Application	Modifiers	Performance		References
				Free Enzymes	Immobilized Enzymes	
**CNT-Ni**	Lipase	Biocatalysis	PyBA4-(1-pyrene)butyric acid	It was inactivated after 40 °C and 24 h	The activity at 40 °C and 24 h was 80.2% of the initial activity	[104]
**CNTs**	Laccase	Biocatalysis for the degradation of phenolic compounds such as hydroquinone	0.3 mol/L HNO_3_ oxidation	The activity was 32% after 50 °C and 4 h	The fixation rate is 96%; the activity was between 54.1% and 84.5% after 50 °C and 4 h	[105]
**CNT**	Lactate dehydrogenase	Biocatalysis	Nitric acid and sulfuric acid	Activity at 70 °C is only 6% of the initial activity	At 70 °C, the activity was 24.0~33.0% of the initial activity	[105]
**MWCNT**	Cellulase	Hydrolyzed cellulase	N-hydroxysuccinimide, APTES	**_**	The fixation rate was 85%, and the enzyme activity remained at 75% after 6 cycles	[106]
**GO**	Pectinase	Food industry	Sodium alginate	15 d completely inactivated	The 15 d enzyme activity was 78.7% of the initial activity	[107]
**GO-NZ**	Laccase	Destaining of the dye	APTES	4 d activity less than 60%	The activity of immobilized laccase remained above 93.0% on the 4th day. The activity of 5 cycles was 95.0% of the initial activity	[108]
**GO**	Lysozyme	Biocatalysis	Sodium alginate	**_**	Maintain the adsorption capacity at 80% after 4 cycles	[109]
**rGO**	Horseradish peroxidase	Environmental governance	**_**	**_**	10 cycles of activity still maintain 70% of the initial activity	[110]
**Fe_3_O_4_NPs**	Lipase	Biocatalyst, green pharmaceutical esters	Chitosan	The 30-day activity is only 31% of the initial activity	The 30-day activity was 95% of the initial activity	[111]
**Fe_3_O_4_**	Laccase	Environmental and catalytic fields	APTES	It is almost inactivated at 25 °C and 20 d	The fixation rate was 76.2~84.4%; cycle 11 times, the activity is about 71%; At 25 °C, the activity of 20 d is about 85%	[112]
**Fe_3_O_4_**	Lysozyme	Enzyme activity studies	Butanetetracarboxylic acid	40 °C, 180 min activity 45.0%; inactivated after 40 days; 49.9% activity at 60	The activity at 40 °C and 180 min was 66.6%. The activity is about 60% after 45 days; the activity at 60 °C was 73.9%; 4 cycles of reuse are still 75% active	[113]
**Fe_3_O_4_@SiO_2_**	Horseradish peroxidase	Environmental remediation and removal of organic pollutants	Polydopamine	The activity was 38.1% at 4 °C and 30 d	70.0% of the activity was recycled for 4 times, and 30.0% was reused for 8 times; the activity was 80.3% at 4 °C and 30 d	[114]

## 5. Vitamin E Succinate

### 5.1. Physical and Chemical Properties and Uses of Vitamin E Succinate

**Figure 2:**
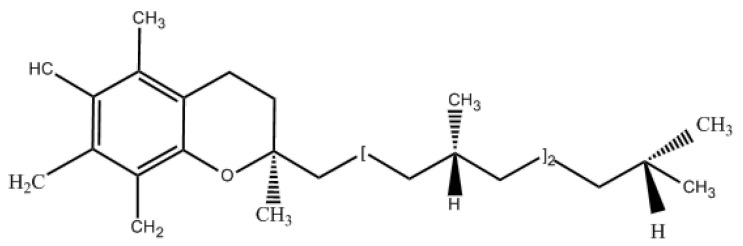
Vitamin E structure.

Vitamin E (tocopherol) is a natural fat-soluble antioxidant that prevents the auto-oxidation of fats and has been widely used in the production of foods such as milk fat, margarine, shortening, powdered oils, emulsifiers, etc. [115]. Vitamin E has a variety of isomers, among which α-vitamin E is the most active and widely distributed, and its chemical structure is shown in Figure 2. Vitamin E contains a phenolic hydroxyl group, but this phenolic hydroxyl group is highly oxidized by air, resulting in a decrease in its biological activity, which severely limits the use of vitamin E in the market. Therefore, the modification of the phenolic hydroxyl group of vitamin E and the synthesis of vitamin E derivatives is an important measure to improve the stability of vitamin E and expand its application.

**Figure 3:**
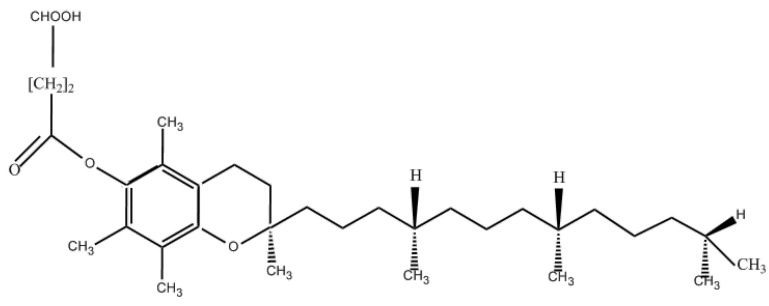
Structural formula of vitamin E succinate.

At present, vitamin E derivatives mainly include vitamin E succinate, acetate, ferulate, nicotinate, and other vitamin E ester products, and these products are widely used in health care drugs, nutritional enhancers, and cosmetic ingredients. As early as 1982, it was established that vitamin E succinate is the most potent form of vitamin E derivative [116]. Currently, vitamin E succinate is also one of the most deeply studied and widely used vitamin E derivatives. Its chemical structure is shown in Figure 3.

The structural difference between vitamin E succinate and vitamin E is only the succinyl group, but its physical and chemical properties are much better than those of vitamin E. Vitamin E succinate effectively expands the field of application of vitamin E and greatly enhances its physiological efficacy. Vitamin E succinate is a solid powder at room temperature, which has good stability, is not easy to be oxidized, and is easy to make health food for tablets and capsules. At the same time, the biological activity of vitamin E succinate gives it unique anti-tumor and anti-cancer functions [18]. Prasad et al. studied the effect of vitamin E succinate on human tumor cells for the first time, and the results showed that vitamin E succinate had an inhibitory effect on the growth of B lymphocytoma cells and significantly increased the concentration of antibodies in the cells. Subsequently, vitamin E succinate has also been shown to inhibit the growth of gastric cancer cells, breast cancer cells, and other malignant tumor cells many times. Studies have shown that the mechanism of anti-tumor action of vitamin E succinate is by blocking the growth cycle of tumor cells, inhibiting DNA synthesis of tumor cells, promoting the secretion of metastatic growth factor β, and enhancing the expression of metastatic growth factor βⅱ receptor can inhibit the proliferation of tumor cells, induce apoptosis of tumor cells, and does not affect the proliferation of normal cells [117,118]. Meantime, vitamin E succinate can also react with calcium hydroxide to produce vitamin E calcium succinate, which has high stability and is easy to prepare tablets and capsules, and can be added to health food as a nutritional booster to supplement vitamin E and calciums [119].

### 5.2. Preparation Method of Vitamin E Succinate

Vitamin E succinate is produced from tocopherol and succinic anhydride by reaction under the action of a catalyst. At present, the main synthesis methods of vitamin E succinate are chemical catalytic synthesis and lipase catalytic synthesis. The catalysts of chemical catalytic synthesis mainly include metals, pyridines, or toxic tertiary amine organic bases (such as triethylamine and tetramethylethylenediamine), which is not in line with people’s advocacy and demand for green chemistry. In addition, the chemical synthesis reaction requires a high temperature, which is easy to oxidize and denature vitamin E, thereby reducing the reaction yield, and the synthesized product is also prone to carbonization to form by-products, which reduces the quality of the product and increases the cost of separation and purification. Compared with the chemical synthesis method, the enzymatic synthesis reaction conditions are mild, the catalytic efficiency is high, and the catalytic specificity is strong. However, the current research on the enzymatic synthesis of vitamin E succinate at home and abroad is not mature enough.

Torres et al. reported for the first time that the enzymatic catalysis of vitamin E acetate was synthesized using vinyl acetate and vitamin E as substrates and using n-hexane and tert-amyl alcohol mixed solvent as reaction media, and the yield of the reaction was only 60% after 18 days, which has no practical application value [120]. Ma et al. developed a strategy using ILs modified lipase to improve the efficiency of vitamin E succinate synthesis [121]. The results show that the CRL modified by 1-butyl-3-methylimidazole and n-acetyl-L-proline ILs has the highest degree of modification, activity and thermal stability. After optimizing the reaction conditions, the yield of vitamin E succinate could be increased to 95.18%. Jiang et al. used natural vitamin E as the reaction substrate and succinic anhydride as the acyl donor, and by screening lipases from different sources and optimizing the reaction conditions, the results showed that the catalytic efficiency of CRL was the highest, and the optimal process conditions were as follows: the molar ratio of the substrate in DMSO was 5:1, the enzyme concentration was 10 g/L, the rotation speed was 100 r/min, and the optimal yield was 46.95% at 55 °C for 18 h [45].

In summary, although the enzyme catalytic synthesis method avoids the shortcomings of the chemical synthesis method of toxic and harmful and many by-products, its catalytic efficiency is not high, and the reaction time and yield are still problems that need to be solved urgently [28]. Therefore, in the enzyme-catalyzed synthesis of vitamin E succinate, it is an effective way to improve the efficiency of enzyme catalysis by selecting enzyme preparations with high activity and good stability and determining the optimal reaction process conditions [122].

## 6. Improvement in Catalytic Performance of Immobilized Lipase and Research Significance and Prospect of Synthesis of Vitamin E Succinate

Starting from the enzyme molecule, through chemical modification, small molecules that can be covalently introduced on the surface of the enzyme molecule for subsequent reaction are introduced, and then the enzyme molecule is embedded in the nanomaterials by aqueous phase in situ polymerization. In situ polymerization can occur between graftase molecules and small monomer molecules, so this method has a higher embedding rate, and fixed more firmly, improve the stability of the immobilized enzyme and organic solvent tolerance.

Vitamin E succinate has a unique pharmacological effect, which enhances the application value of tocopherol and expands the application field of tocopherol. In this paper, CRL nanogel was used as a catalyst to catalyze the synthesis of vitamin E succinate by transesterification reaction in anhydrous DMSO, a “universal solvent” and a strong denaturating agent of protein. The optimum preparation process was studied to improve the yield of the reaction and reduce the production cost of vitamin E succinate.

Lipase is a commonly used and important industrial enzyme, but due to the harsh general industrial production conditions, however, the stability of the free enzyme is poor and difficult to be separated from the product, so its application in industry is greatly limited, so the catalytic performance and stability of lipase can be improved by chemical modification and immobilization. As a new type of immobilized carrier, nanomaterials have the advantages of a large specific surface area and high stability. The results of this study show that the stability of the modified lipase immobilized on the nanomaterial by covalent ligation is significantly improved, and the yield of the modified lipase applied to catalyze the synthesis of vitamin E succinate is also improved, but there are still several aspects that need to be improved.

Lipase was chemically modified with ZIF-8 precursors, and the degree of modification was characterized by X-ray photoelectron spectroscopy (XPS). The results showed that the functional group of the ZIF-8 precursor successfully modified lipase and improved its catalytic activity, but the degree of modification was low. Therefore, it is possible to try more multifunctional organic small molecules to chemically modify lipase to improve the degree of lipase modification, so as to improve the lipase fixed by covalent bond, improve the lipase fixation load, and further improve the stability of immobilized lipase. In addition, some new and mild modification methods can be developed to better improve the activity of lipase.

Ultrasonic-assisted technology can be applied to enzymatic catalysis for the production of vitamin E succinate, and the appropriate frequency and power of ultrasound can also enhance the activity of lipase and increase the reaction speed of enzyme catalysis so as to further reduce the lipase concentration and shorten the reaction time.

During the chemical modification and immobilization of lipase, its enzyme activity and stability have undergone certain changes. Although our group also tried to analyze the structural changes of lipase molecules before and after modification and immobilization by circular dichroism, infrared spectroscopy, and fluorescence spectroscopy. However, the mechanism of the change in the enzymatic properties of lipase cannot be fundamentally explained. Therefore, we can try to add molecular simulation to analyze the modification mechanism of enzymes at the molecular level so as to play a role in rational guidance.

## 7. Conclusions

Summarily, the research progress of enhancing the catalytic performance of immobilized lipase was reviewed, and its application in the synthesis of vitamin E succinate was discussed. The preparation methods of immobilized lipases, including physical and chemical immobilization, and the application of nanomaterials as immobilization carriers were discussed. In addition, the physicochemical properties and application value of vitamin E succinate were also analyzed, and the advantages and disadvantages of its synthesis method were discussed. The results showed that the catalytic activity and stability of lipase could be improved by chemical modification and immobilization so as to increase the yield of vitamin E succinate and reduce the production cost. As a new kind of biocatalyst, immobilized lipase has a broad application prospect in the synthesis of vitamin E succinate. This review provides new ideas and technical support for enhancing the catalytic performance of immobilized lipase and its application in the synthesis of vitamin E succinate.

## Figures and Tables

**Table 1:** Different immobilization methods of enzyme.

Classification	Enzyme Immobilization Method			
	Adsorption	Embedding	Covalent Bonding	Cross-Linking
Advantage	Simple method, Little loss of activity, Cheap and fast	Large amount of immobilized enzyme, No need for extraction or purification, Low loss of activity	Strong bonding properties, Excellent stability	Strongly binds to lipase, Good stability in aqueous solution
Disadvantage	Leaks easily, Binds non-specifically	Methodological complexity, Mass transfer limitations, Leakage	Increased cost, Decreased activity	May be inactive, Lack of mechanical properties, Difficult to control size

## Data Availability

No new data were created or analyzed in this study.
